# Ectomycota Associated with Arthropods from Bat Hibernacula in Eastern Canada, with Particular Reference to *Pseudogymnoascus destructans*

**DOI:** 10.3390/insects7020016

**Published:** 2016-04-22

**Authors:** Karen J. Vanderwolf, David Malloch, Donald F. McAlpine

**Affiliations:** 1 New Brunswick Museum, 277 Douglas Ave, Saint John E2K 1E5, NB, Canada; dmalloch@xplornet.com (D.M.); Donald.McAlpine@nbm-mnb.ca (D.F.M.); 2 Canadian Wildlife Federation, 350 Promenade Michael Cowpland Drive, Kanata K2M 2G4, ON, Canada

**Keywords:** *Pseudogymnoascus destructans*, cave fungi, *Meta ovalis*, *Nelima elegans*, *Scoliopteryx libatrix*, *Exechiopsis* sp., *Anatella* sp., entomopathogens, white-nose syndrome decontamination

## Abstract

The introduction of *Pseudogymnoascus destructans* (*Pd*) to North America, agent of white-nose syndrome in hibernating bats, has increased interest in fungi from underground habitats. While bats are assumed to be the main vector transmitting *Pd* cave-to-cave, the role of other fauna is unexplored. We documented the fungi associated with over-wintering arthropods in *Pd*-positive hibernacula, including sites where bats had been recently extirpated or near-extirpated, to determine if arthropods carried *Pd*, and to compare fungal assemblages on arthropods to bats. We isolated 87 fungal taxa in 64 genera from arthropods. Viable *Pd* was cultured from 15.3% of arthropods, most frequently from harvestmen (*Nelima elegans*). Fungal assemblages on arthropods were similar to those on bats. The different fungal assemblages documented among arthropods may be due to divergent patterns of movement, aggregation, feeding, or other factors. While it is unlikely that arthropods play a major role in the transmission dynamics of *Pd*, we demonstrate that arthropods may carry viable *Pd* spores and therefore have the potential to transport *Pd*, either naturally or anthropogenically, within or among hibernacula. This underlines the need for those entering hibernacula to observe decontamination procedures and for such procedures to evolve as our understanding of potential mechanisms of *Pd* dispersal improve.

## 1. Introduction

The introduction of *Pseudogymnoascus destructans* (*Pd*) to North America, the cause of the fatal disease white-nose syndrome (WNS) in hibernating bats [1], has prompted increased interest in fungi from underground habitats such as caves and mines. WNS has rapidly spread through the eastern United States and Canada, killing >6.7 million bats (an estimate made in 2012) since it was first reported in 2006 in Albany, New York [2]. It is thought that bats are the main vector transmitting *Pd* cave-to-cave within North America [3], but the possible role of other fauna as vectors is largely unexplored. Using culture-independent methods, Lucan, *et al.* [4] found that the external surface of wing mites (*Spinturnix myoti*; removed from *Pd*-positive *Myotis myotis*) were *Pd*-positive (n = 33 mites, 100% positive). Since *S. myoti* are known to switch hosts, Lucan, *et al.* [4] suggest that bat ectoparasites may play a role in the transmission dynamics of *Pd*. Raudabaugh and Miller [5] found that *Pd* grew on autoclaved Migratory Locust (*Locusta migratoria*) in the lab, but it is unclear if *Pd* can compete with the native microflora present on arthropods under field conditions. Additionally, some arthropods in caves are known to produce anti-microbial substances that prevent fungal infection [6]. The microflora on arthropods in caves may differ from that found on bats, and this may also impact the ability of *Pd* to propagate.

The fungi associated with arthropods in caves are relatively well studied compared to other cave fauna; a recent review of fungi in caves listed 201 species of fungi in 89 genera isolated from arthropods, most of which were ascomycetes and zygomycetes [7]. Most studies of fungi on arthropods in caves focus on entomopathogenic fungi [7,8,9], which can reduce populations of cave arthropods. For example *Tolypocladium* sp. occasionally reduces glowworm (*Arachnocampa luminosa*) populations in a New Zealand show cave [10], and the phenomenon of “cricket marshmallows” (*Beauveria caledonica* colonizing *Hadenoecus* spp.) is well known in caves in the United States [11]. Conversely, arthropods consume fungi in caves [12], and may play a role in regulating numbers of cave microfungi [13].

Arthropods may introduce fungi into caves by transporting spores both externally and internally. Cave Crickets (*Ceuthophilus gracilipes gracilipes*) are believed to be vectors for dictyostelid cellular slime molds into and within caves [14]. Entomopathogenic fungal spores can be transmitted among and between insect species, or be acquired from the cave environment [15,16], similar to the hypothesized transmission dynamics for *Pd* [17]. In caves with limited air flow and no rainfall, arthropods may be disproportionally important as spore dispersers. Dickson [18] noted that populations of invertebrates were positively correlated with populations of fungi in Virginia cave sediments. Arthropod exoskeletons possess hairs and crevices that promote spore adherence, and some species of fungi have adaptations for arthropod dispersal, such as spores in sticky drops at the tip of fungal fruiting structures [19]. Outside caves, arthropods are known to be important vectors of fungi that cause plant diseases, such as Dutch Elm disease, transporting viable spores both externally and internally [20].

Fungi associated with hibernating bats in New Brunswick, Canada (*Myotis lucifugus, M. septentrionalis*, and *Perimyotis subflavus*) have been documented over several years [21,22]. WNS was first observed in New Brunswick in March 2011, leading to mass mortality of the hibernating bat population [23]. A diverse assemblage of arthropods have been documented from caves in eastern Canada [24,25], where *Pd* is now widespread. Our objectives in the study reported here were to: (1) determine if viable *Pd* can be cultured from arthropods present in *Pd*-positive mines/caves during the period when bats are hibernating; (2) compare the fungal assemblage from different species of arthropods among mines/caves; and (3) compare the fungal assemblage on arthropods to that found on bats at the same underground sites.

## 2. Materials and Methods

### 2.1. Field Collections

We sampled arthropods in four *Pd*-positive bat hibernacula in New Brunswick: Glebe Mine (abandoned manganese mine) and Dorchester Mine (abandoned copper mine) in March/April 2012–2014, and Markhamville Mine (abandoned manganese mine) and Dallings Cave (limestone) in April 2013. Locations of sites can be found in Vanderwolf, *et al.* [26]. The number of bats present at individual sites were counted during each sampling trip. Visible *Pd*-growth had been observed on hibernating *Myotis lucifugus* and *M. septentrionalis* in Glebe Mine and Dorchester Mine prior to arthropod sampling in 2012, and in Markhamville Mine and Dallings Cave prior to 2013. *Pd*/WNS presence was also confirmed on bats at all sites from samples submitted to the Canadian Wildlife Health Co-operative. Hibernating bats were present during all sampling periods, except in Dorchester Mine in 2013 and Glebe Mine in 2014. Arthropods were sampled ~10–50 m from the entrance of each study site. The mean (± SD) temperature during winter (November 13, 2009 to April 30, 2010) at hibernacula entrances was as follows: Dallings Cave 3.47 ± 2.20 °C, Glebe Mine 1.65 ± 1.90 °C, and Markhamville Mine 1.59 ± 1.47 °C [26]. Dorchester Mine was not studied in 2010. Temperature logger iButtons (model DS1921G, Maxim Integrated Products, Inc., Sunnyvale, CA, USA) were placed in sites and set to record air temperature twice a day (at 0230 and 1430). Two iButtons were deployed within each cave: one in the entrance-twilight zone and the second in the dark zone. A third ibutton was placed above ground at chest height 50–200 m from each hibernaculum entrance. Generally, we sampled arthropods in the same area temperature was recorded, but some sampling also occurred deeper in hibernacula where the temperature was several degrees warmer and more stable than at entrances. We followed the protocol of the United States Fish and Wildlife Service [27] for minimizing the spread of WNS during all visits to caves.

**Figure 1:**
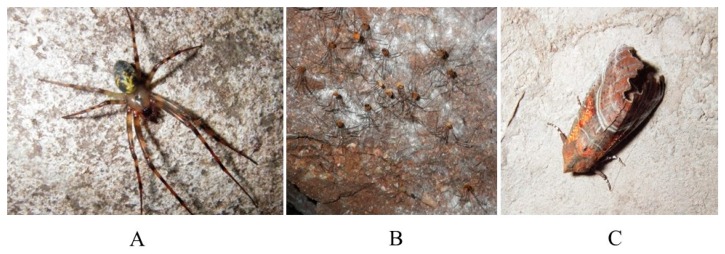
Principal arthropod species in New Brunswick bat hibernacula from which fungi were cultured. (**A**) *Meta ovalis*; (**B**) *Nelima elegans* in loose aggregation; (**C**) *Scoliopteryx libatrix.*

We sampled 17 Cave Orb Weavers (*Meta ovalis* Gertsch 1933), 15 harvestmen (*Nelima elegans* Weed 1889), 17 Herald Moths (*Scoliopteryx libatrix* Linnaeus 1758), and >46 fungus gnats (*Exechiopsis* sp. with a few *Anatella* sp.). Figure 1 shows the three larger arthropod species sampled, with *Nelima elegans* individuals in a “loose aggregation” as defined by Holmberg, *et al.* [28]. Fungus gnats were identified using Vockeroth [29]. Only adult arthropods were sampled. Other arthropods observed at our study sites, including crickets (*Ceuthophilus* spp.), woodlice (*Oniscus asellus*), larger dipterans, crane flies (Tipulidae), and Tissue Moths (*Triphosa haesitata*), were too rare during the study period to provide sufficient sample sizes.

We inoculated two petri plates per individual *M. ovalis, N. elegans*, and *S. libatrix*; one plate with dextrose-peptone-yeast extract agar (DPYA) [30] and the other with Sabouraud-dextrose agar (SAB), both of which were infused with the antibiotics chlortetracycline (30 mg/L) and streptomycin (30 mg/L). These media were previously used to culture fungi from hibernating bats in New Brunswick [21,22]. Pre-poured petri plates were used to directly capture *M. ovalis*, *N. elegans*, and *S. libatrix* from cave walls onto the hardened agar surface, so no individual was handled. Each individual was encouraged to move across the agar surface for ~2 min, during which we ensured that the abdomen touched the agar at least once, before being gently shaken into the next petri dish. Arthropods were then released back onto the cave wall. The media type inoculated first was alternated for each individual arthropod. We captured *Exechiopsis/Anatella* sp. with tweezers sterilized in 95% ethanol and embedded 2–3 individuals directly into the agar surface per plate while in the cave, using equal numbers of both media types. Unlike other arthropods sampled, *Exechiopsis/Anatella* sp. were not removed from the petri plates. All plates were sealed in situ with parafilm.

### 2.2. Laboratory Methods

In the laboratory, samples were incubated in a low-temperature incubator (Model 2015, VWR International, Mississauga, ON, Canada) in the manner reported previously (inverted, in the dark at 7 °C) for studies of fungi associated with bats hibernating at the same sites [21,22]. Samples were monitored over 4 months until either no new cultures had appeared for 3 weeks on a plate, or plates had become overgrown with hyphae. Once fungi began growing on plates, each distinct colony was sub-cultured to a new plate. DPYA without oxgall and sodium propionate was used for maintaining pure cultures.

Identifications were accomplished by comparing the micro- and macro-morphological characteristics of microfungi to those traits appearing in the taxonomic literature and compendia [31,32]. Identifications were also made by comparison to an existing reference collection of fungal cultures from *Myotis lucifugus* and *M. septentrionalis* that were previously identified in 2010 using a mix of sequencing and morphological features [21]. Identifications of *Pd* were confirmed by sequencing as part of other studies ([33] for 2012 samples; [34] for 2012, 2013, and 2014 samples). Permanent cultures are housed in the University of Alberta Microfungus Collection and Herbarium (UAMH 11726-11728), and desiccant-dried samples are in the New Brunswick Museum (NBM# F-04966–05026, 05028–05038, 05144–05150, 05156–05160, 05162, 05170–05173, 05175, 05177–05182, 05184–05198; 05351–05358, 05360–05362, 05371–05393, 05401, 05426–05427, 05435–05454, 05559, 05627, 05639–05640). We totaled the number of fungal taxa per individual arthropod specimen, and determined the number of individuals of each arthropod species that each fungal taxon was identified from.

### 2.3. Statistical Analysis

The number of fungal taxa/individual arthropod were square root transformed and compared amongst sites, years, and arthropod species with a general linear model and Tukey post-hoc tests. Using previously assembled data on fungi from over-wintering bats (*Myotis lucifugus, M. septentrionalis*, and *Perimyotis subflavus*) in Glebe Mine [21,22], the number of fungal taxa/individual were square root transformed and compared amongst arthropod and bat species from Glebe Mine using a 1-way ANOVA with a Tukey post-hoc test. The number of times each fungal taxon was isolated was compared amongst arthropod species with a Kruskal-Wallis test. The number of fungal taxa obtained from the first plate inoculated was compared to the second plate for *N. elegans, M. ovalis*, and *S. libatrix* using a paired t-test after the data were square root transformed. A Mann-Whitney test was used to compare the number of fungal taxa obtained per plate for DPYA versus SAB. All statistical analysis were performed using Minitab (Minitab Inc., State College, PA, USA).

## 3. Results

Fungi were cultured from 71 of 72 arthropods sampled (counting each plate of *Exechiopsis/Anatella* sp. as an individual) and from 114 of 119 (95.8%) field-inoculated plates. This produced a total of 559 isolates. One *N. elegans* escaped before the second plate could be completed. A mean of 6.6 ± 3.8 fungal taxa were isolated from each arthropod (Table 1). The mean number of fungal taxa/individual arthropod was not significantly different across years (F_2,71_ = 2.24, *p* = 0.115), but was significantly higher on *Nelima elegans* and *Scoliopteryx libatrix* when compared to *Meta ovalis* and *Exechiopsis/Anatella* sp. (F_3,71_ = 17.98, *p* < 0.001, Table 1). The mean number of fungal taxa/individual arthropod was significantly higher in Glebe Mine when compared to the other three sites (F_3,71_ = 12.21, *p* < 0.001). Within Glebe Mine, the mean number of fungal taxa/individual was significantly higher on *N. elegans* when compared to *Myotis lucifugus*, *Meta ovalis*, and *Exechiopsis/Anatella* sp., and significantly lower on *Exechiopsis/Anatella* sp. compared to *Perimyotis subflavus* (F_6,48_ = 5.10, *p* = 0.001; Table 1).

During this study, 87 fungal taxa in 64 genera plus 20 sterile fungal morphs were isolated from four arthropod species (Table 2). Thirty-seven (42.5%) of the 87 fungal taxa were isolated from a single individual arthropod each. Thirty-one fungal taxa were isolated from *Exechiopsis/Anatella* sp. (n = ~46 individuals, 23 plates), 33 from *M. ovalis* (n = 17 individuals, 34 plates), 46 from *S. libatrix* (n = 17 individuals, 34 plates), and 53 from *N. elegans* (n = 15 individuals; 29 plates). Viable *Pd* was cultured from 15.3% of arthropods (n = 72), with 26.7% of *N. elegans*, 17.7% of *M. ovalis*, 17.4% of *Exechiopsis/Anatella* sp., and 0% of *S. libatrix Pd*-positive (Table 2). *Pd* was isolated from 16.0% of arthropods in 2012 (n = 25), 27.3% in 2013 (n = 22), and 4.2% in 2014 (n = 24; Table 3). These figures do not show consistent trends with the number of hibernating bats present during sampling or year-to-year temperature variation (Table 3). We did culture viable *Pd* from some arthropods in hibernacula from which bats had been extirpated or near-extirpated in the years prior to sampling. The most common fungal taxa were *Cladosporium* spp. (isolated from 52.8% of arthropods; n = 72), *Penicillium* spp. (45.8%), *Mortierella* spp. (38.9%), *Verticillium* sp. *cf. Gabarnaudia* (36.1%), *Acremonium* spp. (33.3%), *Mucor* spp. (31.9%), *Cephalotrichum stemonitis* (29.2%), and *Leuconeurospora polypaeciloides* (29.2%). The number of times each fungal taxon was isolated from arthropods was significantly different among arthropod species (H_3,347_ = 12.55, *P* = 0.006). However, when fungal taxa that occurred ≤3 times were excluded, the difference was not significant (H_3,115_ = 5.02, *P* = 0.17). Therefore, common fungal taxa occurred at similar frequencies on all four arthropod species, while uncommon fungi differed amongst arthropod species. Some fungi appeared to be associated with specific arthropod species more often than others, such as *Botrytis* sp., *Fusarium* sp., and *Phoma* sp. on *S. libatrix* and *Acrodontium* spp. on *N. elegans* (Table 2). The lack of *Cladosporium* spp. on *M. ovalis* is also notable. Visible fungal growth on arthropods was not observed during our sampling period.

The most common fungal taxa isolated from bats in Glebe Mine were *Cephalotrichum stemonitis* (isolated from 84.21% of bats, n = 19; data taken from [21,22]), *Leuconeurospora polypaeciloides* (68.42%), *Baeospora* sp. (63.16%), *Humicola cf*. UAMH 11595 (63.16%), *Wardomyces* spp. (63.16%), *Preussia* sp. (47.37%), *Mortierella* spp. (42.11%), and *Penicillium* spp. (36.84%). The most common fungal taxa isolated from arthropods in Glebe Mine were *Penicillium* spp. (isolated from 70.00% of arthropods, n = 30), *Cephalotrichum stemonitis* (63.33%), *Cladosporium* spp. (53.33%), *Leuconeurospora polypaeciloides* (53.33%), *Mortierella* spp. (43.33%), *Acremonium* spp. (40.00%), *Mucor* spp. (36.67%), and *Verticillium* sp. *cf. Gabarnaudia* (36.67%). Some fungal taxa occurred more often or exclusively on bats, such as *Baeospora* sp. (63.2% on bats, n = 19; 0% on arthropods, n = 30), unidentified Basidiomycetes (36.8%, 10%), *Humicola cf.* UAMH 11595 (63.2%, 26.7%), and *Preussia* sp. (47.4%, 13.3%). Other fungal taxa occurred more often or exclusively on arthropods, such as *Verticillium* sp. *cf. Gabarnaudia* (36.7% on arthropods, 0% on bats), *Botrytis* sp. (10%, 0%), *Acrodontium* sp. (16.7%, 0%), *Phoma* sp. (20%, 5.3%), *Acremonium* spp. (40.0%, 10.5%), *Penicillium* spp. (70.0%, 36.8%), *Cladosporium* spp. (53.3%, 5.3%), *Hormonema* sp. (10%, 0%), and *Mucor* spp. (36.7%, 5.3%).

A total of 65 fungal taxa were isolated from arthropods in Glebe Mine whereas 50 fungal taxa were cultured from arthropods in Dorchester Mine. The composition of the most common fungal taxa were similar between the two sites, but Glebe Mine had a greater diversity of fungi that were isolated on a single occasion compared to Dorchester Mine. In particular, higher numbers of *Cephalotrichum stemonitis, Leuconeurospora polypaeciloides, Wardomyces* spp., and *Humicola cf.* UAMH 11595 were isolated from Glebe Mine (63.33%, 53.33%, 26.67%, and 26.67% of arthropods, respectively, n = 30) compared to Dorchester Mine (2.94%, 8.82%, 0%, and 5.88%, n = 34).

DPYA produced 301 isolates (n = 58 plates) while SAB produced 258 isolates (n = 61 plates), but the number of isolates per plate was not significantly different between the two media types (W_1,118_ = 3767, *p* = 0.125). The first plate inoculated for *N. elegans, M. ovalis*, and *S. libatrix* did not have a significantly higher fungal diversity than the second plate (T_1,71_ = −0.89, *p* = 0.382). The second plate contributed a mean of 2.08 ± 1.83 fungal taxa from *M. ovalis* which were not detected with the first plate. Likewise, 4.08 ± 2.31 fungal taxa on *N. elegans*, and 2.42 ± 2.19 fungal taxa cultured from *S. libatrix* were detected with the second plate but not the first. *Pd* was isolated from six SAB plates and five DPYA plates, but tended to appear on the first plate used, regardless of agar type (5 on first plate, 2 on second plate).

## 4. Discussion

Viable *Pd* was isolated from *Nelima elegans, Meta ovalis*, and *Exechiopsis/Anatella* sp., but not *Scoliopteryx libatrix*. *Nelima elegans* appear to acquire a greater diversity of fungal spores when compared to the other arthropod species sampled. This may be one reason why harvestmen produced the highest *Pd* yield. *Nelima* spp. are known to overwinter in the twilight zone of caves in eastern and southwestern Canada from October to May. Although this species may form aggregations of up to tens of thousands [25,28,35], we most commonly observed groups of 2–15. Harvestmen are thought to aggregate in caves to optimize their immediate microclimate, particularly to maintain exposure to high humidity [28]. However, aggregation may also promote the acquisition and transmission of fungi, including *Pd*. As a mobile species *N. elegans* may encounter organic matter in caves (a source of fungal spores; [36]) more frequently than other arthropods. Some harvestmen species have been observed consuming fungi growing on wood in caves [37]. *Acrodontium* spp. were particularly abundant on *N. elegans* compared to other arthropods sampled during this study, and preliminary ITS sequencing indicate that at least three *Acrodontium* species were isolated (Keith Seifert, Agriculture Canada, per. comm.). *Acrodontium* spp. have been found in soil, air, and on mites and spiders [38,39], and some species are considered plant pathogens [40]. *Acrodontium* sp. have been isolated from soil in Antarctica [41], indicating that some species in the genus are cold tolerant. Previous studies of fungi on harvestmen in caves are few: Meyer-Rochow and Liddle [42] observed *Metarrhizium anisopliae* growing on dead *Megalopsalis tumida* in a cave in New Zealand and Holmberg, *et al.* [28] noted unidentified fungi growing on dead *Leiobunum paessleri* in caves in British Columbia, Canada. Machado, *et al.* [43] reported that *Goniosoma longipes* were frequently infected with unidentified fungi in caves in Brazil. Outside caves a diversity of entomopathogenic fungi have been isolated from a variety of harvestmen species, including *Asplenium trichomanes, Cordyceps gonylepticida, Engyodonthium aranearum, Entomophthora phalangicida, Hymenostilbe verrucosa, Metarrhizium anisopliae, Nomuraea atypicola, Pandora phalangicida,* and *Phyllactinia guttata* [42,44,45,46,47,48,49]. *Entomophaga batkoi* was described from harvestmen and is known to cause considerable mortality in European harvestmen, with epizootics observed during late summer [50,51]. Harvestmen produce exocrine secretions that may act as fungicides [48], which may enable them to cope with high diversities of fungi.

Although *S. libatrix* is believed to be largely sedentary during the winter, when this species occupies hibernacula, *S. libatrix* nonetheless carried a relatively diverse fungal assemblage. Why *Pd* was not cultured from *S. libatrix* is unclear. Unlike *N. elegans*, over-wintering *S. libatrix* do not aggregate in large numbers, although groups of 2–6 and 2–11 have been observed in caves in Manitoba [52] and Poland [53]. During the present study, most *S. libatrix* roosted individually near or on the ceiling with groups of 2–3 rarely observed. This isolation may decrease the transmission of fungi, including *Pd*, when compared to aggregating species such as *N. elegans*. Additionally, over-wintering *S. libatrix* rarely move and do not feed in hibernacula. Combined, these behaviors may limit the number of spores moths encounter. In caves in Manitoba and Ontario, *S. libatrix* were not observed moving in or out of caves, but numbers increased in the autumn and declined in spring [52,54]. Movement of individuals within caves was limited to a few cm over the winter, with the majority of individuals found within 10m of the cave entrance near the ceiling [52]. In Poland, Kowalski [53] noted greater within-cave movements (up to 10m) of over-wintering *S. libatrix* in caves subject to rapid temperature shifts, and occasionally observed *S. libatrix* just outside the hibernation site. Of the arthropods examined during this study, *S. libatrix* has the greatest potential of moving spores cave-to-cave. Individual moths can hibernate for two successive winters in the same cave [52]. *Scoliopteryx libatrix* produces two generations a year and individuals of the first generation appear in June and fly together with those which have hibernated [53]. Previous studies of fungi on *S. libatrix* in caves have focused on moth cadavers, with unidentified fungi observed growing on cadavers in caves in Poland [53] and Manitoba, Canada [52]. In caves in the Czech Republic, *Engyodontium rectidentatum, Lecanicillium muscarium, Paecilomyces farinosus* (most common), *P. fumosoroseus, Simplicillium cf. lamellicola*, and 2 sterile morphs were cultured from 30 dead *S. libatrix* [55]. Aside from *Engyodontium*, these genera were also isolated from live *S. libatrix* in the current study. *Phoma* sp., *Botrytis* sp., and *Fusarium* sp., which are generally considered plant pathogens [31], were isolated more frequently from *S. libatrix* in New Brunswick compared to the other three arthropod species sampled and may have been transported into hibernacula on the moths.

*Meta ovalis* do not aggregate and individuals are generally found 12–14 cm from each other, but can occasionally be found in close proximity (1–5 cm) [56]. Rector [56] found little movement of *M. ovalis* within caves, and no movement between caves. While this may limit the number of fungal spores spiders encounter, clearly it does not prevent acquisition of *Pd* spores by *M. ovalis*. Yoder, *et al.* [9] noted that *M. ovalis* generally appear inactive, but can occasionally be found outside caves. Spiderlings of a closely related European species, *M. menardi*, disperse from natal underground sites in spring and return to the same site in late summer or find new sites via ballooning [57]. Therefore, spiderlings may be more important than adults in spreading fungi cave-to-cave. As observed during this study, *M. ovalis* occurs most commonly in the entrance and twilight zones near or on the ceiling, but may sometimes be encountered in deeper parts of caves [9,56]. *Meta ovalis* may acquire spores from other arthropods caught in spider webs. Spider webbing outside caves is known to trap fungal spores, including species of *Alternaria, Cladosporium*, and *Fusarium*, among other Ascomycota and Basidiomycota [58], with this potentially contributing to the fungal diversity found on the external surface of spiders. Yoder, *et al.* [9] sampled 40 freshly killed *M. ovalis* in a Kentucky cave and commonly cultured *Aspergillus* sp., *Mucor* sp., *Penicillium* sp., and *Rhizopus* sp., with fewer occurrences of *Absidia* sp., *Beauveria* sp., *Cladosporium* sp., *Paecilomyces* sp., *Trichoderma* sp., and sterile morphs. The low fungal diversity Yoder, *et al.* [9] reported from *M. ovalis* compared to the present study is likely due to the shorter incubation times followed by these authors (5–10 days), which would favor the fast-growing fungi they documented.

The overall low fungal diversity we found associated with *Exechiopsis/Anatella* sp. may be due to the small body size and short life span of these dipterans compared to the other arthropods and bats we sampled. Fungus gnat adults live days to weeks, while adult moths, harvestmen, and spiders survive months to years [53,59,60]. Although fewer plates were inoculated with *Exechiopsis/Anatella* sp., a greater number of individuals were sampled. Fungi associated with fungus gnats in caves have not been previously studied, but Keates, *et al.* [61] sampled fungus gnats (*Bradysia* sp.) in conifer nurseries in British Columbia and cultured species of *Penicillium, Cladosporium, Mucor, Rhizopus, Cephalosporium, Alternaria, Ulocladium, Fusarium, Botrytis cinerea*, and *Phoma*. Fungi associated with an insect of similar size to the fungus gnat, the mosquito (*Culex pipiens*), have been studied in caves. Teernstra-Eeken and Engel [62] sampled *C. pipiens* and Heleomyzidae flies in caves in the Netherlands and cultured *Lecanicillium lecanii, Beauveria bassiana, Isaria farinosa, Aspergillus* sp., *Polycephalomyces formosus, Hirsutella entomophila, Hirsutella saussurei, Paecilomyces* sp., and *Entomophthora* sp. (most common). These fungi, largely *Entomophthora* sp., caused 56%–97% mortality of overwintering *C. pipiens*, with the highest mortality in February, just prior to the emergence of *C. pipiens* from caves [62]. In caves in Czechoslovakia, over-wintering *C. pipiens* had 85% mortality due to infection with *Entomophthora destruens* [63]. Although we did culture *Pd* from *Exechiopsis/Anatella* sp. with a frequency very similar to that of *M. ovalis*, the short life-span, small body size, and perhaps limited dispersal ability of fungus gnats, probably limits the possible role of these dipterans in transporting *Pd* spores within or between caves.

Eighty-nine fungal genera have previously been documented from arthropods in caves, most commonly *Beauveria, Aspergillus, Laboulbenia, Penicillium, Rhachomyces, Mucor, Mortierella, Cladosporium, Paecilomyces, Lecanicillium, Isaria*, and *Hirsutella* [7]. Many of these genera are known entomopathogens and are targeted by investigators, particularly *Laboulbenia* and *Rhachomyces*, suggesting their relative abundance on arthropods in the cave environment may be overestimated. Entomopathogens, although present, were not commonly isolated during this study, which was carried out during the winter months when temperatures are low. Entomopathogens, such as species of *Tolypocladium, Paecilomyces, Beauveria*, and *Isaria*, have optimal growth temperatures of 20–30 °C [64]. However, some, such as *Beauveria bassiana*, are able to grow at temperatures as low as 5 °C, albeit at a slow rate [65]. Eilenberg, *et al.* [66] found that the entomophthoralean fungus *Entomophthora schizophorae* survives the winter in its host, the adult dipteran *Pollenia* sp., through slow disease development and transmission among hosts hibernating in clusters in unheated attics. Overwintering near entrances and in the twilight zone of caves and mines, where temperatures are relatively low, can reduce metabolism and hence extend food reserves in harvestmen and other arthropods [28]. In addition, arthropods may avoid fungal infections where they overwinter close to the entrance of cold, northern hibernacula. Overwintering arthropods are known to venture deeper into caves [56] where temperatures are higher than at entrances, but may be more susceptible to fungal infections in these warmer temperatures. Higher relative humidity, as found in the dark zone of caves compared to the light zone, increases fungal survival, germination, and sporulation, as well as facilitating penetration of insect cuticles by entomopathogenic fungi [67,68]. We have previously isolated entomopathogens from bats in these hibernacula [21,22], confirming their presence in the dark zone at our study sites.

The composition and number of fungal taxa per individual was similar when comparing arthropods to bats at the same hibernaculum. Other studies have found a higher diversity of fungi closer to cave entrances compared to the dark zone [69,70]. However, this pattern was not apparent at our sites when arthropods from the cave entrance were compared to bats hibernating in the dark zone. Currently there is little information on how individual arthropod or bat species interact with sources of fungal spores in the environment, however, many of these influences are probably site-specific. For example, we found that bats hibernating in Glebe Mine harbored greater numbers of basidiomycetes, particularly *Baeospora* sp., than arthropods at the same site. This may be because bats roosted deeper within the mine, where *Baeospora* sp. and other mushrooms were observed growing on wood, while arthropods overwintered in the entrance-twilight zone where mushrooms were absent. Several of the fungal genera that were more common on arthropods than on bats, such as *Verticillium* sp., *Botrytis* sp., *Hormonema* sp., and *Phoma* sp., are often associated with green plants and may have been blown into the hibernaculum entrance, where arthropods are concentrated, or transported underground from the surface on the arthropods themselves.

Although arthropods and bats over-winter in different areas of underground hibernacula, arthropods are in the flight paths of bats arriving and leaving caves and mines. Furthermore, during autumn swarming, bats may roost or alight at cave entrances. Arthropods may therefore acquire *Pd* spores from cave walls where fungal spores have been shed from passing bats. However, the growth of *Pd* at cave entrances, whether in the environment or on arthropods themselves, cannot be ruled out at this time and could contribute to the long-term persistence of *Pd* in caves in the absence of bats [71]. *Pd* can grow at temperatures as low as 0.8 °C, albeit more slowly than at its optimum growth temperature of 12–15 °C [72]. The variation in the proportions of *Pd*-positive arthropods over the course of this study did not seem to be related to year-to-year temperature variation. The highest yields of *Pd* did not occur during the warmest winters. The proportion of *Pd*-positive arthropods decreased from 2012 to 2014 in Glebe Mine as the over-wintering bat population declined due to WNS-related mortality. This suggests that bats are a source of *Pd* spores at hibernacula entrances. However, we did not observe such a decrease in Dorchester Mine, where the number of *Pd*-positive arthropods peaked in 2013, despite the extirpation of over-wintering bats (from a high of 140 *M. lucifugus* and *M. septentrionalis* in 2011) due to WNS mortality. Our sampling of *N. elegans*, the arthropod species with the highest yield of *Pd*, in Dorchester Mine in 2012 and 2014 was limited compared to 2013 (1 in 2012, 6 in 2013, and 2 in 2014), and this may partly explain the pattern. Results suggest a reservoir of viable *Pd* spores remained in the environment, available for transmission to arthropods, for at least a year following extirpation, or near-extirpation, of the overwintering bat population. Likewise, we recorded *Pd*-positive arthropods at Glebe Mine in 2014, when hibernating bats were no longer present. While the data presented here suggests it is unlikely that arthropods play a major role in the transmission dynamics of *Pd*, we demonstrate that arthropods may carry *Pd* spores and therefore have the potential to transport *Pd* within or among hibernacula should they disperse naturally or be moved anthropogenically from *Pd*-positive sites. The latter underlines the need for those entering hibernacula to observe accepted decontamination procedures and for such procedures to evolve as our understanding of the potential mechanisms of *Pd* dispersal improve. Although natural dispersal of arthropods from hibernacula is unlikely to occur over great distances, it does appear that the movement of *Pd* spores via arthropods from specific caves or mines can potentially continue to occur for at least a year after the extirpation of overwintering bat populations.

## 5. Conclusions

We isolated 87 fungal taxa in 64 genera from four species of arthropods overwintering in eastern Canadian bat hibernacula. These sites were occupied, or formerly occupied, by bats infected with the fungus *Pseudogymnoascus destructans*, the cause of white-nose syndrome. Viable *Pd* was cultured from 15.3% of arthropods up to a year after extirpation or near-extirpation of resident bat populations and most frequently from harvestmen (*Nelima elegans*). Fungal assemblages on arthropods were similar to those on bats in the same sites. While it is unlikely that arthropods play a major role in the transmission dynamics of *Pd*, we demonstrate that arthropods may carry viable *Pd* spores and therefore have the potential to transport *Pd,* either naturally or anthropogenically, within or among hibernacula. This underlines the importance for those entering hibernacula to observe decontamination procedures and for such procedures to evolve as our understanding of potential mechanisms of *Pd* dispersal improve.

## Figures and Tables

**Table 1:** The mean number (± standard deviation) of fungal taxa per individual arthropod (per plate for *Exechiopsis/Anatella* sp.) over three years in three mines and one cave in New Brunswick, Canada.

Arthropod & Bat Species	Glebe Mine	Dorchester Mine	Markhamville Mine	Dallings Cave	All sites	Range
*Meta ovalis*	7.0 ± 1.9 (8) ^BC^	2.8 ± 2.2 (9)	ND	ND	4.76 ± 2.95 (17) *	0–10
*Scoliopteryx libatrix*	9.7 ± 4.3 (6) ^ABC^	8.4 ± 2.5 (5)	6.0 ± 1.4 (2)	6.5 ± 4.2 (4)	8.12 ± 3.59 (17) ^&^	2–15
*Nelima elegans*	13.6 ± 3.0 (5) ^A^	8.3 ± 3.1 (9)	7 (1)	ND	10.00 ± 3.89 (15) ^&^	4–18
*Exechiopsis/Anatella* sp.	5.8 ± 1.8 (11) ^C^	3.3 ± 1.2 (11)	ND	ND	4.55 ± 1.97 (23) *	1–9
*Perimyotis subflavus*	10.2 ± 2.2 (9) ^AB^	ND	ND	ND	ND	7–14
*Myotis lucifugus*	7.7 ± 3.7 (6) ^BC^	ND	ND	ND	ND	2–12
*Myotis septentrionalis*	9.3 ± 4.0 (4) ^ABC^	ND	ND	ND	ND	6–15
All arthropod species	8.2 ± 3.8 (30)	5.2 ± 3.4 (34)	6.3 ± 1.2 (3)	6.5 ± 4.2 (4)	6.61 ± 3.78 (72)	0–18

The number of arthropods sampled are in parentheses. Means from Glebe Mine that do not share a letter are significantly different (*p* < 0.001). Pooled means from all sites with different symbols (*, ^&^) are significantly different from each other (*p* < 0.001). The range is the number of fungal taxa per individual (per plate for *Exechiopsis/Anatella* sp.) across all sites. Data from *Myotis lucifugus*, *M. septentrionalis,* and *Perimyotis subflavus* taken from Vanderwolf, *et al.* [21,22] are included for comparison. ND = no data.

**Table 2:** Fungal taxa isolated from the external surface of over-wintering arthropods in caves and mines in New Brunswick, Canada over three years (2012–2014). Column figures indicate the number of individuals (plates for *Exechiopsis/Anatella* sp.) culturing positive for each fungal taxon. Gl = Glebe Mine, Do = Dorchester Mine, Ma = Markhamville Mine, and Da = Dallings Cave.

Fungal Taxon	*Meta ovalis*		*Scoliopteryx libatrix*				*Nelima elegans*			*Exechiopsis/Anatella sp.*		
	Gl	Do	Gl	Do	Ma	Da	Gl	Do	Ma	Gl	Do	all
**Ascomycota**												
*Acremonium* sp.	2	0	3	4	0	1	3	6	0	4	1	24
*Acremonium* sp. (hyaline)	0	0	0	0	0	0	0	1	0	0	0	1
*Acrodontium* spp.	0	0	1	0	0	0	4	9	0	0	2	16
*Alternaria* sp.	0	0	1	0	0	0	0	0	0	0	0	1
*Aureobasidium* sp.	0	0	0	0	0	0	0	1	0	0	0	1
*A. pullulans* (De Bary) G. Arnaud ex Cif., Ribaldi & Corte	0	0	1	0	0	0	0	0	0	0	0	1
*Beauveria bassiana* (Bals.-Criv.) Vuill.	0	0	0	0	0	0	0	0	0	0	2	2
*Beauveria* sp. (penicillate)	0	0	1	0	0	0	0	0	0	0	0	1
*Botrytis* sp.	0	0	3	3	1	1	0	1	0	0	0	9
*Cephalotrichum stemonitis* (Pers.) Link	8	0	1	1	0	0	2	0	1	8	0	21
*Chaetomidium* sp.	0	0	0	0	0	0	0	1	0	0	0	1
*Cladosporium* sp.	0	0	6	5	2	4	5	4	1	4	7	38
*C. cladosporioides* complex (Fresen.) G.A. de Vries	0	0	0	0	0	1	0	0	0	0	0	1
*cf. Conioscypha* sp.	0	1	0	0	0	0	0	0	0	0	0	1
*cf. Cylindrocarpon* sp.	0	0	0	1	0	0	0	0	0	0	0	1
*Dactylella* sp.	1	0	0	0	0	0	0	0	0	0	0	1
*Dendryphiella* sp.	0	1	0	0	0	0	0	0	0	0	0	1
*Exophiala* sp.	0	0	1	0	0	1	0	1	0	0	0	3
*Fusarium* sp.	0	0	2	1	0	2	0	0	0	0	0	5
*Fusicladium cf. carpophilum* (Thum.) Oudem	1	0	0	0	0	0	0	0	0	0	0	1
*Hormonema* sp.	0	0	2	0	0	0	1	0	0	0	0	3
*Humicula cf.* UAMH 11595	2	1	1	0	2	0	2	0	1	3	1	13
*Hyalodendriella* sp.	0	0	0	0	0	0	1	0	0	0	0	1
*Isaria farinosa* (Holmsk.) Fr.	0	0	1	2	0	0	0	0	0	0	0	3
*Lecanicillium* sp.	0	0	0	0	0	0	0	0	0	0	2	2
*L. muscarium* (Petch) Zare & W. Gams	0	0	1	0	0	0	0	1	0	0	0	2
*Leuconeurospora capsici* (J.F.H. Beyma) Malloch, Sigler & Hambleton	0	0	0	0	0	1	0	0	0	3	1	5
*L. cf. pulcherrima* (G. Winter) Malloch & Cain	0	0	0	0	0	0	0	1	0	0	0	1
*L. polypaeciloides* Malloch, Sigler & Hambleton	4	1	1	0	1	0	4	1	1	7	1	21
*Malbranchea* sp.	1	0	0	0	0	0	0	0	0	0	0	1
*Mammaria* sp.	0	0	0	0	0	0	0	0	0	1	0	1
*Microascus* sp.	1	0	0	0	0	0	0	0	0	0	0	1
*M. caviariformis* Malloch & Hubart	0	0	0	0	0	0	0	0	0	0	1	1
*M. cf. giganteus* Malloch	1	0	1	0	0	0	1	0	0	0	0	3
*Monodictys* sp.	0	0	0	0	0	0	1	0	0	0	0	1
*Myceliophthora* sp.	1	0	0	0	0	0	1	0	0	0	0	2
*Oidiodendron* sp.	0	0	0	0	0	0	0	0	0	1	0	1
*O. truncatum* G.L. Barron	1	1	1	0	0	0	0	2	0	0	0	5
*Paecilomyces* sp.	0	0	0	2	0	0	1	0	0	0	0	3
*P. inflatus* (Burnside) J.W. Carmich.	0	0	0	0	0	0	0	3	0	0	0	3
*cf. Penicillifer* sp.	0	0	0	0	0	0	1	0	0	0	0	1
*Penicillium* sp.	4	2	3	4	0	2	2	5	0	5	2	29
*P. cf. brevicompactum* Dierckx	0	0	0	0	1	0	0	0	0	0	0	1
*P. cf. decumbens* Thom	0	0	0	0	0	0	1	0	0	0	0	1
*P. cf. soppii* K.M. Zalessky	0	0	0	0	0	1	0	0	0	0	0	1
*P. cf. thomii* Maire	0	0	2	1	0	0	3	0	0	0	1	7
*Phaeotrichum hystricinum* Cain & M.E. Barr	1	0	0	0	0	0	1	0	0	1	1	4
*Phialemonium* sp.	0	0	0	0	0	0	0	1	0	0	0	1
*Phoma* sp.	0	0	4	3	2	2	2	0	0	0	0	13
*Preussia* sp.	2	0	0	0	0	0	1	0	0	1	0	4
*Pseudogymnoascus destructans* (Blehert & Gargas) Minnis & D.L. Lindner	1	2	0	0	0	0	0	4	0	4	0	11
*P. pannorum* (Link) Minnis & D.L. Lindner *senso lato*	5	1	1	2	0	2	4	0	0	3	2	20
*Sarocladium strictum* (W. Gams) Summerbell	0	0	1	0	0	0	0	0	0	0	0	1
*Scopulariopsis* sp.	0	0	0	0	0	1	0	1	0	0	0	2
*Simplicillium* sp.	0	0	1	2	0	0	1	1	0	0	0	5
*Stachybotrys* sp.	0	0	0	0	0	0	0	0	0	0	1	1
sterile	1	4	3	2	0	1	2	4	0	1	2	20
*Streptomyces* sp.	0	0	0	0	0	0	0	0	0	0	1	1
*Thelebolus* sp.	1	0	0	0	0	0	0	0	0	0	0	1
*Thelebolus crustaceus* (Fuckel) Kimbr.	0	0	0	0	0	0	1	0	1	0	0	2
*Thysanophora* sp.	0	0	1	0	0	0	0	0	0	0	0	1
*T. penicilliodes* (Roum.) W.B. Kendr.	0	2	1	0	0	0	2	4	0	1	1	11
*Tolypocladium cf. cylindrosporum* W. Gams	0	0	0	0	0	0	1	0	0	0	0	1
*T. inflatum* W. Gams	1	0	0	0	0	0	0	1	0	0	1	3
*Trichoderma* sp.	0	0	0	0	0	1	0	1	0	0	0	2
*Trichosporiella* sp.	0	0	0	0	0	0	1	1	0	1	0	3
*Verticillium* sp.	0	0	0	2	0	1	1	1	0	0	0	5
*Verticillium cf. alboatrum* Reinke & Berthold	0	0	1	1	0	0	0	0	0	0	0	2
*Verticillium* sp. (*cf. Gabarnaudia*)	4	6	1	1	0	0	2	7	0	4	1	26
*Wardomyces* sp.	0	0	0	0	0	0	0	0	0	1	0	1
*W. cf. columbinus* (Demelius) Hennebert	0	0	0	0	0	0	0	0	0	1	0	1
*W. humicola* Hennebert & G.L. Barron	1	0	0	0	0	0	0	0	0	0	0	1
*W. inflatus* (Marchal) Hennebert	1	0	0	0	0	0	2	0	0	2	0	5
*Zopfiella pleuropora* Malloch & Cain	1	0	0	0	0	0	1	0	0	0	0	2
*Zythia* sp.	0	0	0	1	0	0	0	0	0	0	0	1
**Basidiomycota**												
*Asterotremella* sp.	0	0	0	0	0	0	3	0	0	1	0	4
Basidiomycete unidentified	0	1	2	0	0	0	1	0	0	0	0	4
*Cystofilobasidium* sp.	1	0	0	0	0	0	0	0	0	0	0	1
*Trichosporon* sp.	1	0	1	0	1	0	2	1	0	0	0	6
*T. dulcitum* (Berkhout) Weijman	1	0	0	0	1	0	0	0	0	0	0	2
**Zygomycota**												
*Mortierella* sp.	3	1	3	1	0	1	3	8	1	4	3	28
*Mucor* sp.	4	0	1	3	2	3	2	2	1	4	1	23
*Thamnidium elegans* Link	0	0	1	0	0	0	1	0	0	0	0	2
*Umbelopsis isabellina* (Oudem.) W. Gams	0	0	1	0	0	0	1	0	0	0	0	2

**Table 3:** The percentage of arthropods in bat hibernacula in New Brunswick, Canada from which *Pseudogymnoascus destructans* (*Pd*) was cultured over three years (2012–2014).

Bat Hibernacula	2012			2013			2014		
	*Pd* yield	# of bats	Temperature	*Pd* yield	# of bats	Temperature	*Pd* yield	# of bats	Temperature
Glebe Mine	30% ($\frac{1}{3}$M, $\frac{3}{6}$A, $\frac{0}{3}$S)	174	5.83 ± 1.48 ^a^ (D)	0% ($\frac{0}{2}$M, $\frac{0}{2}$N)	22	5.98 ± 1.94 (D)	7.1% ($\frac{0}{2}$M, $\frac{0}{2}$N, $\frac{1}{4}$A, $\frac{0}{2}$S)	0	5.33 ± 2.09 (D) 0.06 ± 1.33 ^b^ (E) −2.67 ± 7.66 (O)
Dorchester Mine	0% ($\frac{0}{3}$M, $\frac{0}{1}$N, $\frac{0}{7}$A, $\frac{0}{2}$S)	1	6.63 ± 0.00 ^c^ (D) 1.08 ± 2.67 ^c^ (E) −0.97 ± 7.12 ^c^ (O)	54.5% ($\frac{2}{4}$M, $\frac{4}{6}$N, $\frac{0}{1}$S)	0	6.59 ± 0.12 (D) −2.19 ± 6.91 ^c^ (O)	0% ($\frac{0}{3}$M, $\frac{0}{3}$N, $\frac{0}{5}$A, $\frac{0}{3}$S)	2	6.61 ± 0.07 (D) −3.93 ± 7.29 ^c^ (O)
Markhamville Mine	ND		5.33 ± 0.52 ^a^ (D)	0% ($\frac{0}{1}$N, $\frac{0}{2}$S)	16	5.28 ± 0.37 (D)	ND		4.89 ± 0.69 (D) −0.64 ± 2.68 (E) −3.71 ± 7.69 (O)
Dallings Cave	ND		3.55 ± 1.29 ^d^ (D)	0% ($\frac{0}{4}$S)	2	4.26 ± 2.07 (D) −0.12 ± 6.03 (O)	ND		3.59 ± 2.26 (D) 0.84 ± 3.20 (E) −2.96 ± 7.79 (O)

Species of arthropods are in parentheses with the number of *Pd*-positive individuals (plates for dipterans) over total individuals sampled. M = *Meta ovalis*; N = *Nelima elegans*; A = *Exechiopsis/Anatella* sp.; S = *Scoliopteryx libatrix.* The number (#) of bats (*Myotis lucifugus*, *M. septentrionalis*, and *Perimyotis subflavus*) present during sampling is indicated, although bats roosted deeper within sites than arthropods. The mean temperature ± SD in °C at the entrance (E), deep (D), and just outside (O) of sites is listed. Temperatures were taken Nov 1 to Apr 30 except where indicated. Missing temperature values indicate failed ibuttons. ND = no data. ^a^ Nov 30 to Apr 30; ^b^ Dec 5 to Apr 30; ^c^ Dec 8 to Apr 30; ^d^ Dec 16 to Apr 30.

## Abbreviations

The following abbreviations are used in this manuscript:

*Pd*: *Pseudogymnoascus destructans*
WNS: white-nose syndrome
DPYA: dextrose-peptone-yeast extract agar
SAB: Sabouraud-dextrose agar
